# Genome-wide analysis of transcription factor binding sites and their characteristic DNA structures

**DOI:** 10.1186/1471-2164-16-S3-S8

**Published:** 2015-01-29

**Authors:** Zhiming Dai, Dongliang Guo, Xianhua Dai, Yuanyan Xiong

**Affiliations:** 1 Department of Electronics and Communication Engineering, School of Information Science and Technology, Sun Yat-Sen University, Guangzhou 510006, China; 2 State Key laboratory for Biocontrol, Sun Yat-Sen University, Guangzhou 510275, China; 3 SYSU-CMU Shunde International Joint Research Institute, Shunde, China

## Abstract

**Background:**

Transcription factors (TF) regulate gene expression by binding DNA regulatory regions. Transcription factor binding sites (TFBSs) are conserved not only in primary DNA sequences but also in DNA structures. However, the global relationship between TFs and their preferred DNA structures remains to be elucidated.

**Results:**

In this paper, we have developed a computational method to generate a genome-wide landscape of TFs and their characteristic binding DNA structures in *Saccharomyces cerevisiae*. We revealed DNA structural features for different TFs. The structural conservation shows positional preference in TFBSs. Structural levels of DNA sequences are correlated with TF-DNA binding affinities.

**Conclusions:**

We provided the genome-wide correspondences of TFs to DNA structures. Our findings will have implications in understanding TF regulatory mechanisms.

## Background

Proper control of gene expression is critical for the complex function of a living cell. Although gene expression can be regulated at multiple levels, one of the most important regulatory mechanisms is at the transcriptional level. The transcriptional program is dependent on binding of transcription factors (TFs) to the cis-acting regulatory elements in promoter and enhancer regions of genes. Transcription factors also regulate gene expression by recruiting coactivators and RNA polymerase II (RNA Pol II) to target genes [1]. TFs and their binding sites are thus fundamental to the regulation of gene expression.

TFs bind DNA in a sequence-specific manner. Binding sites of one TF share conserved (i.e. similar) primary sequence patterns in different target promoters. The conserved sequence patterns have been widely used to computationally identify transcription factor binding sites (TFBSs) [2-5]. However, the traditional one-dimensional view of DNA sequence is oversimplified. The three-dimensional structure of DNA, which reflects the physicochemical and conformational properties of DNA, is critical for the packaging of DNA in the cell [6]. The structure of DNA has been recognized to be important for protein-DNA recognition [7,8].

DNA bending plays a role in the regulation of prokaryotic transcription [9]. DNA structure can be used as discriminatory information to identify core-promoter regions [10,11]. Specific replication-related proteins show a preference to bind curved DNA sequences [12]. DNA curvature is also involved in the binding of recombination-related proteins to DNA [13]. DNA structure in the human genome is more evolutionary constrained than the primary nucleotide sequence alone [14]. Moreover, the DNA structure-conserved regions correlate with non-coding regulatory elements, better than sequence-conserved regions identified solely on the basis of primary sequence [14].

Although primary nucleotide sequences determine three-dimensional structures of DNA, different DNA sequences might have similar DNA structures, one TF might bind DNA with different primary sequence patterns but with similar DNA structures. Recently, several computational approaches have used DNA structural properties to identify TFBSs with modest success [15-20]. There are many DNA structural properties that potentially influence TF-DNA binding. Different TFs might prefer different DNA structural properties. However, the full relationship between TFs and their corresponding DNA structural properties remains to be elucidated. In this study, we evaluated DNA structure in terms of various physicochemical and conformational properties. We have developed a computational approach to derive the first genome-wide landscape of TFs and their featured binding DNA structures in budding yeast *Saccharomyces cerevisiae*. We found that a considerable number of TFs have distinct DNA structural preferences. These structural features show positional preferences in TFBSs.

## Results

### A compendium of DNA structural properties

We used 35 types of di- or trinucleotide DNA structural properties, which were mainly collected in our previous study [21]. The structural properties chosen in this study have been frequently used and have been extensively studied in previous literatures [22,23]. These structural properties provide important information on the structure of DNA and capture structural properties that might be of importance for transcription. Each property contains complementary information and provides a unique insight into the DNA structure. The properties were classified into two types: conformational and thermodynamic. The rationale for exploiting di- or trinucleotide properties is the widely accepted nearest neighbor model saying that DNA structure can be understood and caused largely by interactions between neighboring base pairs [24,25]. This model is typically in the form of dinucleotide or trinucleotide properties. Each possible di- or trinucleotide and its reverse complement are assigned with a parametric value for a single structural property. The origins of the parametric values are either derived from experimentally determined structures, or from simulated structures of a DNA helix or a DNA-protein complex.

### Construction of the landscape of TFs and their characteristic binding DNA structures

**Figure 1:**
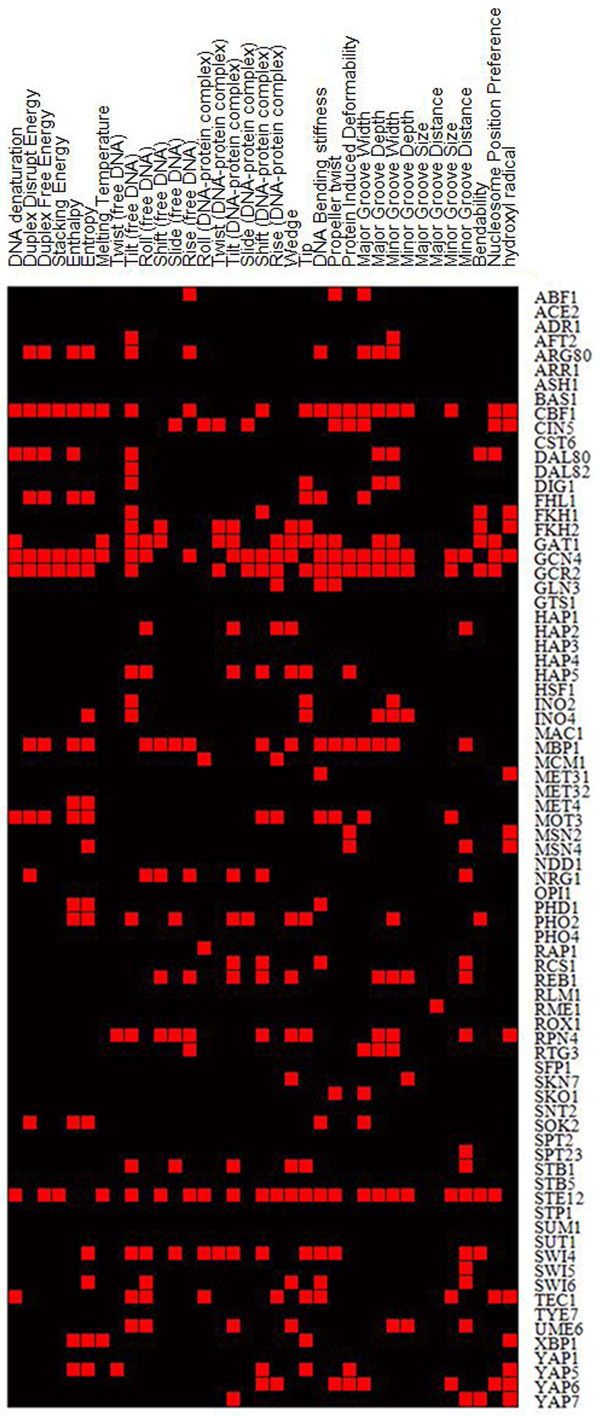
**The landscape of TFs and their characteristic binding DNA structures**. Rows represent TFs, and columns represent DNA structures. For each TF-structure pair, if structural conservation rate of its real TFBSs is significantly higher (*P *< 0.05, after Bonferroni correction for multiple testing) than those in 10,000 randomized experiments in which sequence conservation rates are the same as that of real TFBSs, it was colored red, otherwise it was colored black.

We examined whether TFs show a preference to bind sequences with specific DNA structures. To this end, we examined whether binding sites of one particular TF are conserved in some DNA structures. We used genome-wide experimentally measured 6,390 TFBSs for 118 TFs in *S. cerevisia *[26]. We restricted the analysis to TFs with more than 15 binding sites, resulting in 77 TFs. For each TF, we calculated the conservation rate in DNA structures of its TFBSs for each of the 35 DNA structural properties (see Materials and Methods). DNA structure is dependent on DNA sequence. As TFBSs are known to be conserved in DNA primary sequences, this might bias the conservation of TFBSs in DNA structures. We should control conservation in DNA sequences when evaluating conservation in DNA structures. The conservation of TFBSs in DNA sequences could be measured by the information content (IC) of position weight matrices (PWMs) of TFBSs [27]. For each TF, we randomly generated a set of TFBSs from its real PWM, the number of which is the same as the number of its real TFBSs. The PWM of randomly generated TFBSs is the same as real PWM, so the conservation in DNA sequences of randomly generated TFBSs is the same as that of real TFBSs. We generated 10,000 randomized sets of TFBSs for each TF. For each set of TFBSs, we also calculated the conservation rate in DNA structure for each of the 35 DNA structural properties. For each TF, we calculated *p*-value for each structural property according to the ranking of its real conservation rate in those of 10,000 randomized sets. We found that 50 out of 77 (~65%) TFs bind DNA sequences that are significantly conserved in at least one structural property (ranging from one to twenty-six structural properties, a total of 356 pairs of TF-structure correspondences) (*P *< 0.05, after Bonferroni correction for multiple testing; Figure 1). This result indicates that a considerable number of TFs bind DNA sequences that show conservation in distinct DNA structures, independent of conversation in DNA sequences.

**Figure 2:**
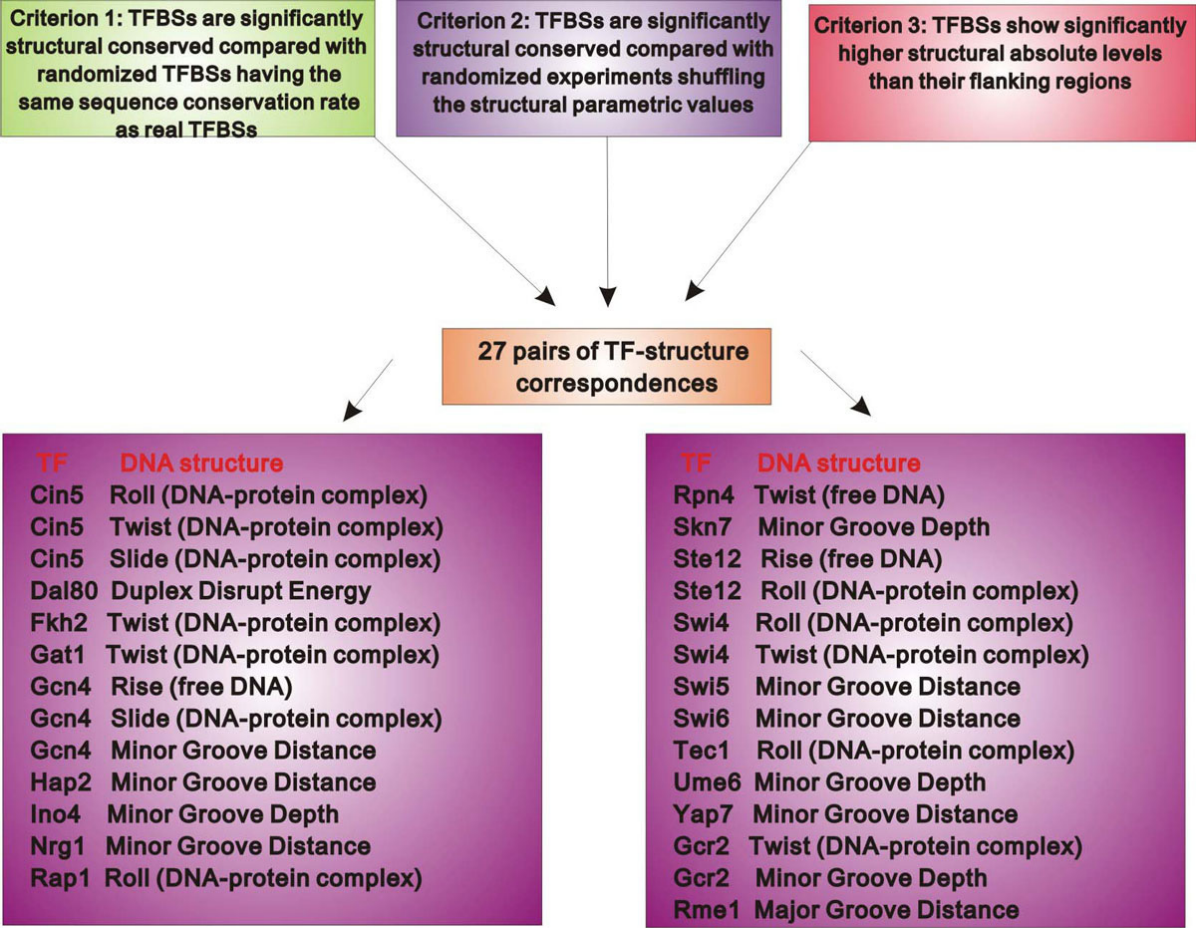
**The refined landscape of TFs and their characteristic binding DNA structures**. Using three criteria, we identified 27 pairs of TF-structure correspondences. TFBSs of these TFs are conserved in the corresponding DNA structures, independent of sequence conservation.

We next filtered the above landscape of TF-structure correspondences using more criteria. First, for each structural property, we randomly shuffled the parametric values among the di- or trinucleotides. We generated 10,000 randomized profiles for each structural property. For each TF, we calculated the conservation rates in DNA structures of its TFBSs as above based on these randomized profiles. For each TF, we calculated *p*-value for each structural property according to the ranking of its real conservation rate in those of 10,000 randomized profiles. If the 356 TF-structure pairs observed above is not an artifact, the real structural conversation rates of TFBSs should be significantly higher than those based on the randomized structural profiles. 39 out of the 356 TF-structure pairs show significantly higher conservation rates in the corresponding structures (*P *< 0.05, after Bonferroni correction for multiple testing). Second, the apparent conservation of TFBSs in DNA structures might be biased by the DNA structures of flanking regions around TFBSs. If TFBSs show similar DNA structural levels as their flanking regions, the conservation of TFBSs in DNA structures should be considered as an artifact. For the 39 pairs of TF-structure correspondences, we found 27 pairs whose TFBSs show significantly higher absolute levels in the corresponding structures than their flanking regions (from -30 to +30 bp relative to TFBS) (*P *< 0.05, after Bonferroni correction for multiple testing). Together, we used three strict criteria to generate 27 pairs of TF-structure correspondences (Figure 2). We used these 27 TF-structure pairs in the following study unless otherwise stated.

The 27 TF-structure pairs observed above demonstrate the characteristic associations between TFs and DNA structures of their binding sites. We found that there is selectivity of TFs and DNA structures involved in the associations: 20 of the 77 TFs examined show associations with DNA structures, and 9 of the 35 DNA structures examined are connected with TF binding (Figure 2). Furthermore, some specific TFs are associated with more DNA structures than the other TFs. There are two TFs (Cin5 and Gcn4) that are associated with three DNA structures.

### Structural conservation shows positional preferences in TFBSs

**Figure 3:**
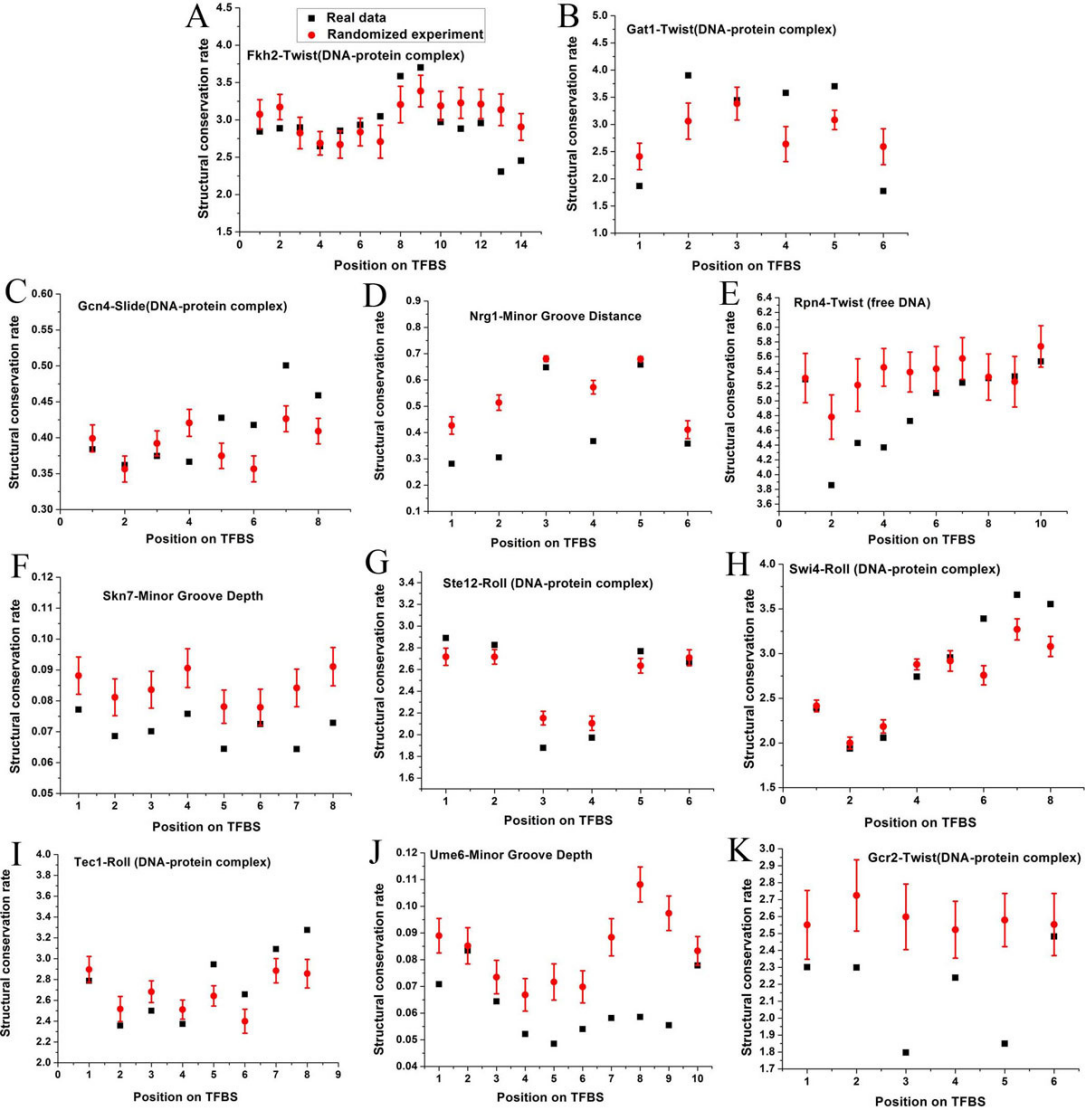
**Structural conservation shows positional preferences in TFBSs**. Real conservation rates of structures are shown for each position of TFBSs (black). Low levels correspond to high conservation rates. Average conservation rates of structures in 10,000 randomized experiments in which TFBSs are generated from real PWMs are also shown (red). Error bars were calculated by standard deviation. The names of TF-structure correspondences are also indicated. TFs show significantly higher conservation in their corresponding structures than those based on 10,000 randomized experiments in the following specific positions in TFBSs (*P *< 0.05, after Bonferroni correction for multiple testing): (A) The thirteenth and fourteenth positions; (B) The first and sixth positions; (C) The fourth position; (D) The first, second, third, fourth and fifth positions; (E) The second, third, fourth and fifth positions; (F) The first, second, third, fourth, fifth, seventh and eighth positions; (G) The third and fourth positions; (H) The third and fourth positions; (I) The third position; (J) The first, fourth, fifth, sixth, seventh, eighth and ninth positions; (K) The second, third, fourth and fifth positions.

We asked whether TFs-associated structural conservation rates are homogeneous along TFBSs. To this end, we compared DNA structural conservation rate of each position in TFBSs with those in 10,000 randomized experiments. As above, we used the random TFBSs generated from real PWMs. 11 out of 20 TFs listed in Figure 2 show significantly higher conservation in their correspondent structures in specific positions of TFBSs than those based on 10,000 randomized experiments (*P *< 0.05, after Bonferroni correction for multiple testing; Figure 3). The binding sites of most TFs show significantly higher structural conservation in more than one specific positions. The binding sites of two TFs, including Ste12 and Swi4, show significantly higher structural conservation in two successive positions. For example, conservation of roll property in the third and fourth positions of TF Ste12 binding sites (Figure 3G). These results suggest that DNA structures of some specific positions in TFBSs might be more important for the binding of TFs to DNA. For example, using an extensive categorization of the biophysical structures of TF DNA-binding domains [28,29], we found that Rap1 and Tec1, having the helix-turn-helix domains, show a preference to bind DNA sequences that are conserved in roll structural property.

### TF-DNA binding affinities are correlated with DNA structural levels of binding sequences

**Figure 4:**
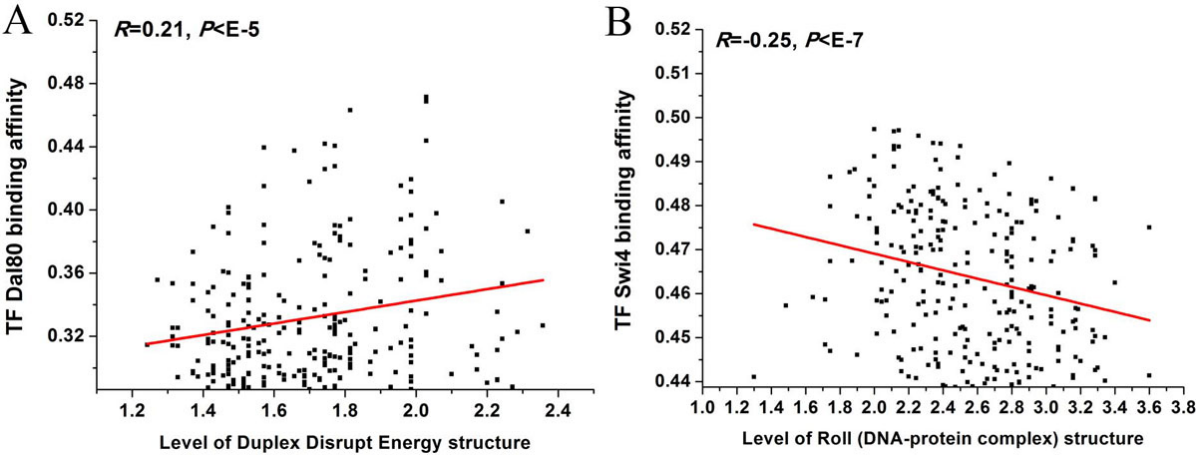
**Structural levels of DNA sequences are significantly correlated with TF-DNA binding affinities**. (A) Shown is a scatter plot comparison between structural (duplex disrupt energy) levels of 8-mers and TF Dal80 binding affinities of 8-mers. To control for nonspecific protein-DNA binding, we restricted the analysis to the top 500 out of 65,536 8-mers with the highest Dal80 binding affinities. The Pearson correlation and the ***p***-value of the scatter plot are indicated. The duplex disrupt energy property of DNA sequences facilitates Dal80 binding to DNA. (B) Same as (A), but for TF Swi4 and structure roll. To control for nonspecific protein-DNA binding, we restricted the analysis to the top 500 out of 65,536 8-mers with the highest Swi4 binding affinities. The roll property of DNA sequences inhibits Swi4 binding to DNA.

We asked whether TF-DNA binding affinities are correlated with DNA structures of binding sequences. A previous study has integrated binding affinities of 153 yeast TFs to all 8-bp sequences (8-mers) (*N *= 65,536) *in vitro *utilizing protein-binding microarray (PBM) [30]. We used this data instead of *in vivo *data because *in vivo *TF-DNA binding is influenced by many factors besides TFBS, including nucleosome positioning, histone modification and so on. PBM data [30] is available for 14 out of 20 TF listed in Figure 2. For each 8-mer, we calculated its structural level for each of the 35 structural properties. We found that binding affinities of 10 out of 14 TFs to DNA are significantly correlated with their correspondent structural levels of DNA sequences (Pearson correlation coefficient, |R| > 0.1, *P *< 0.05; see selected examples in Figure 4). These results suggest that our observed TF-associated structures play a role in TF binding.

## Discussion

In this study, we performed a systematic analysis to reveal the relationship between TFs and their preferred DNA structures. Using three strict criteria, we found that a considerable number of TFs bind DNA sequences that are structurally conserved, independent of sequence conservation in *S. cerevisiae*. Moreover, we found that the structural conservation of TFBSs is also prevalent in other eukaryotes (unpublished data). These three strict criteria are very important to ensure a low level of false positives. However, some TFs do not show association with DNA structure. It does not indicate that DNA structure is not important to binding of these TFs to DNA. First, structural conservation of TFBSs might be largely determined by sequence conservation, so that structural conservation could not be detected when controlling for sequence conservation. Second, TFBSs of these TFs might be conserved in some unknown DNA structures. Advances in structural biology will give more insights into structures of TFBSs.

A key finding of this study is that structural conservation shows positional preference in TFBS. As our analysis is controlled for sequence conservation, the positional preference of structural conservation is not an artifact of the positional preference of sequence conservation. This finding could tell which position in TFBS is more important to TF-DNA binding. The local structure determined by these positions is more critical for TF-DNA recognition. The change in these local structure is more likely to influence TF-DNA binding and subsequent TF regulation. More attention should be paid to these local structures when analyzing cancer cell lines. It also will have implication in synthetic biology. It might help to distinguish functional TFBSs from non-functional TFBSs. On the other hand, some TFs whose binding sites are structurally conserved do not show structural positional preference. The binding of these TFs to DNA might be dependent on the DNA structure of the whole TFBS.

Despite its success, our approach has limitations. TFs generally interact with different protein factors to regulate target genes. These protein factors might influence the conformation of TFs, changing TF binding preference. TFs with similar DNA-binding domains might show different structural preferences for binding of DNA. One TF might even show different structural preferences for different target genes due to its different protein partners. Our method might miss this type of TF-structure correspondence.

## Materials and methods

### Calculation of DNA structural conservation rate

We used 35 types of conformational and thermodynamic DNA di- or trinucleotide structural properties, which were used in our previous study [21] (see Additional file 1 for more details about each of these structural properties), as measures of DNA structure. For a DNA region, the sequence is divided into overlapping di- or trinucleotide sequences. Structural profiles from DNA sequences are calculated for each structural property (except for hydroxyl radical cleavage pattern) as follows: The corresponding parametric value for each di- or trinucleotide was assigned to the first nucleotide of the di- or trinucleotide. In this way, the nucleotide sequence is converted into a sequence of numbers (i.e., a numerical profile). For hydroxyl radical cleavage intensity data, structural profiles are calculated as the reference where the data was published [31]. The hydroxyl radical cleavage intensity data are assigned to each nucleotide in each trinucleotide sequence. Note that the three nucleotides in each trinucleotide sequence have different values of hydroxyl radical cleavage intensity. As each nucleotide (except for the two terminal nucleotides at each end of the DNA region) is covered by three overlapping trinucleotide sequences, it has three values of hydroxyl radical cleavage intensity (one for each trinucleotide). The three values are averaged to produce hydroxyl radical cleavage intensity for each nucleotide. In this way, the nucleotide sequence is converted into a sequence of numbers (i.e., a numerical profile). For each region, the average of its numerical profile is considered as the level of the corresponding structure. For each pair of regions (e.g. TFBSs), we calculated the absolute difference values of structural profiles. For each TF, we calculated absolute difference profiles of structural profiles between every possible pairs of TFBSs (Additional file 2). We considered the average of resulting absolute difference profiles normalized by the length of TFBSs as a measure of conservation rate of DNA structure. The low values correspond to high conservation rates. In this way, there were 35 measures of structural conservation rate for TFBSs of each TF. Similarly, we also calculated absolute difference value of structural profiles at each position between every possible pairs of TFBSs, and then calculated conservation rate of DNA structure at each position of TFBS.

### Data preparation

Transcription factor binding data was taken from MacIsaac et al. [26]. A *p-*value cutoff of 0.005 and conservation among three species was used to define the sequence bound by a particular TF. By applying this strict binding threshold, we ensured a low level of false positives. The data set includes 6,390 binding sites for 118 TFs. We mapped binding sites to the corresponding genes according to their located promoters (600 bp upstream of the gene in this study, the upstream region was truncated if it overlapped with neighboring genes). If the binding sites locate between divergent gene pairs, we mapped the binding sites to their nearest genes.

Gene coordinate data and genome sequence were downloaded from the Saccharomyces Genome Database [32]. TF binding affinity data for 8-mers were taken from Gordân et al.[30]. TF classification data were downloaded from two literatures [28,29].

### Statistical method

Given two samples of values, the Mann-Whitney U-test is designed to examine whether they have equal medians. The main advantage of this test is that it makes no assumption that the samples are from normal distributions.

## Competing interests

The authors declare that they have no competing interests.

## Authors' contributions

ZD and DG implemented the algorithms and carried out the experiments. ZD also designed the study, analyzed the results and drafted the manuscript. DG, XD and YX participated in the analysis and discussion. All authors read and approved the final manuscript.

## Supplementary Material

Additional file 1 **Table S1 **List of dinucleotide/trinucleotide DNA structural properties and their corresponding parameters Click here for file

Additional file 2 **Figure S1 **An example of how to calculate absolute difference profiles of structural profiles between one pair of TFBSs. For each TF, we calculated absolute difference profiles of structural profiles between every possible pairs of TFBS. We considered the average of resulting absolute difference profiles normalized by the length of TFBSs as a measure of conservation rate of DNA structure. Click here for file

## Declarations

The research and publication has been supported by National Natural Science Foundation of China (NSFC) (Grant 61202343), by Natural Science Foundation of Guangdong Province (S2012040007935), and also by Fundamental Research Funds for the Central Universities (Grant 13lgpy06).

This article has been published as part of *BMC Genomics *Volume 16 Supplement 3, 2015: Selected articles from the 10th International Symposium on Bioinformatics Research and Applications (ISBRA-14): Genomics. The full contents of the supplement are available online at http://www.biomedcentral.com/bmcgenomics/supplements/16/S3.
