# Supplementary figures and images for: Genome-wide analysis of transcription factor binding sites and their characteristic DNA structures
Source: BMC Genomics. 2015 Jan 29;16(Suppl 3):S8. doi: 10.1186/1471-2164-16-S3-S8 (PMC4331811; doi:10.1186/1471-2164-16-S3-S8)

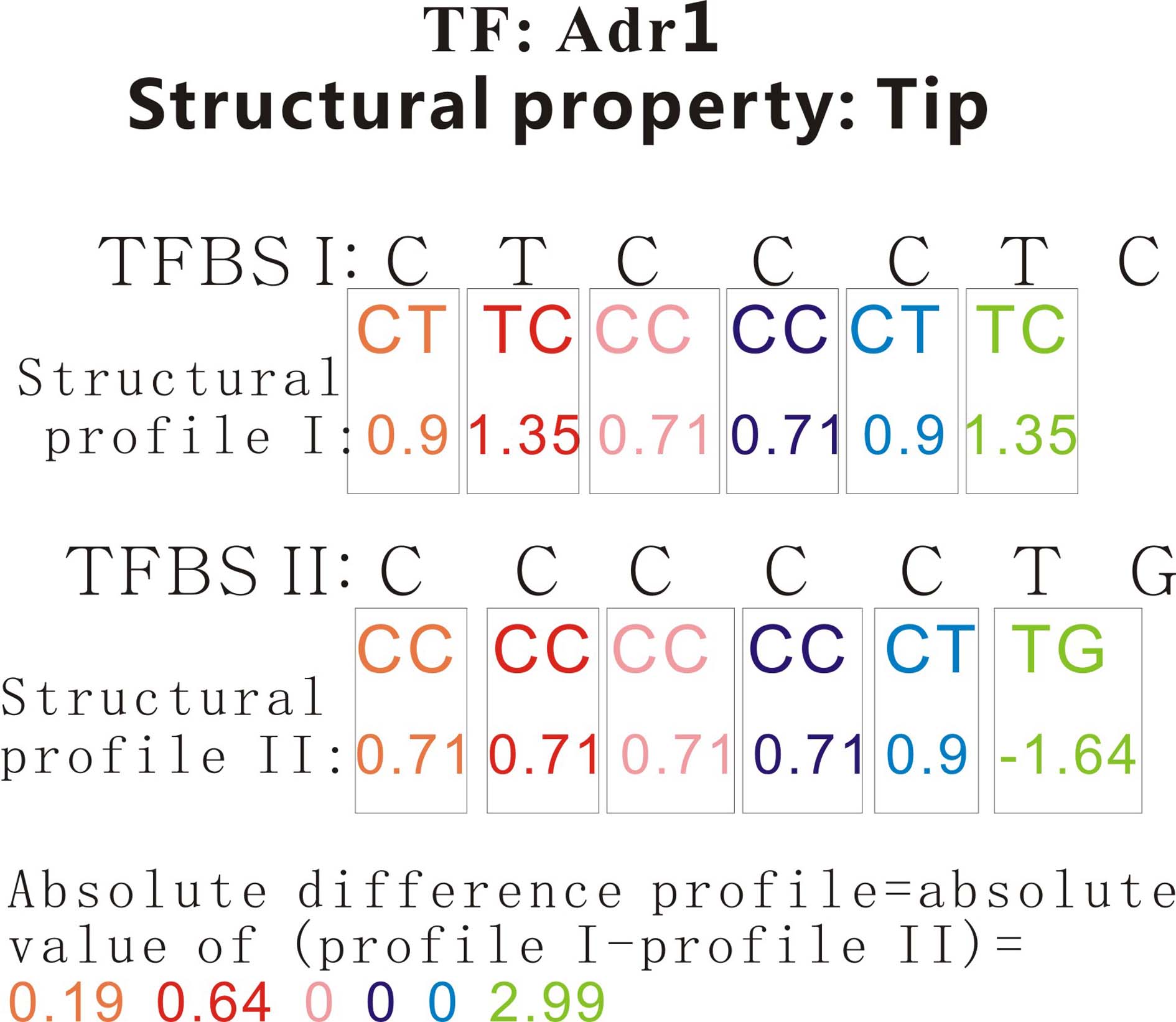

Supplement: Additional file 2 — Figure S1 An example of how to calculate absolute difference profiles of structural profiles between one pair of TFBSs. For each TF, we calculated absolute difference profiles of structural profiles between every possible pairs of TFBS. We considered the average of resulting absolute difference profiles normalized by the length of TFBSs as a measure of conservation rate of DNA structure. [file 1471-2164-16-S3-S8-S2.jpg]
